# Toll-like receptor 5-mediated IL-17C expression in intestinal epithelial cells enhances epithelial host defense against F4^+^ ETEC infection

**DOI:** 10.1186/s13567-019-0665-8

**Published:** 2019-06-20

**Authors:** Yu Luo, Jia Xu, Chaoying Zhang, Chunyan Jiang, Yanfeng Ma, Haijian He, Yuan Wu, Bert Devriendt, Eric Cox, Hongbin Zhang

**Affiliations:** 1 Animal Medical Testing Center, Department of Animal Production, Faculty of Agricultural & Biological Engineering, Jinhua Polytechnic, Jinhua, China; 2 Laboratory of Immunology, Faculty of Veterinary Medicine, Ghent University, Ghent, Belgium

## Abstract

Enterotoxigenic *Escherichia coli* (ETEC) are an important cause of post-weaning diarrhea (PWD) in piglets. The IL-17 cytokine family is well known to play important roles in the host defense against bacterial infections at the mucosa. Previously, we reported the potential role of IL-17A in clearing an ETEC infection in piglets. IL-17C, another member of the IL-17 family, is highly expressed in the intestinal epithelium, however, its role during an ETEC infection is still unclear. In this study, we demonstrate that F4^+^ ETEC induce IL-17C mRNA and protein expression in intestinal tissues as well as in porcine intestinal epithelial cells (IPEC-J2). This IL-17C production is largely dependent on TLR5 signaling in IPEC-J2 cells. Both F4^+^ ETEC infection and exogenous IL-17C increased the expression of antimicrobial peptides and tight junction proteins, such as porcine beta-defensin (pBD)-2, claudin-1, claudin-2 and occludin in IPEC-J2 cells. Taken together, our data demonstrate that TLR5-mediated IL-17C expression in intestinal epithelial cells enhances mucosal host defense responses in a unique autocrine/paracrine manner in the intestinal epithelium against ETEC infection.

**Electronic supplementary material:**

The online version of this article (10.1186/s13567-019-0665-8) contains supplementary material, which is available to authorized users.

## Introduction

The importance of the IL-17 cytokine family in inflammation and autoimmunity is well recognized. This family consists of six members: IL-17A, IL-17B, IL-17C, IL-17D, IL-17E (also called IL-25) and IL-17F [1]. These cytokines bind to heterodimeric complexes composed of members of the IL-17 family of receptors: IL-17 receptor (IL-17R) A, IL-17RB, IL-17RC, IL-17RD, and IL-17RE, to elicit their biological effects [2]. IL-17A and IL-17F have been well studied and have been shown to be mainly expressed by a distinct T cell subset, T helper type 17 cells. IL-17A/F initiates innate host defenses and repair responses that include the induction of proinflammatory cytokines and chemokines, antimicrobial peptides and proteins, such as β-defensin, calprotectin and cathelicidins [3]. In contrast to IL-17A/F, the biological function of IL-17C and the molecular mechanisms regulating IL-17C expression are less well investigated. Similar to IL-17A and IL-17F, IL-17C also seems to mediate inflammatory processes and has been detected in lung and skin tissues after bacterial infection as well as in the colon of inflammatory bowel disease patients [4–6]. Notably, IL-17C and its receptor IL-17RE are preferentially expressed in epithelial cells lining the mucosa and play an essential role in the host mucosal defense against microbial infection by triggering the production of chemokines, inflammatory mediators as well as antimicrobial peptides [7–9]. Despite its potential role in inflammation and host defense, the function of IL-17C in pigs remains poorly understood.

F4^+^ enterotoxigenic *E. coli* (ETEC) is one of the most common causes of diarrhea in neonatal and recently weaned piglets [10, 11], resulting in considerable morbidity and mortality. Intestinal epithelial cells (IECs) are the first cells encountering intestinal pathogens and not only form a physical barrier preventing passage of macromolecules and pathogens to the underlying tissues, but also sense and detect pathogen-associated molecular patterns (PAMPs) through pathogen-recognition receptors (PRRs), such as Toll-like receptors (TLRs), to initiate the innate immune response [12]. Porcine IECs have been shown to express several TLRs and to enhance cytokine mRNA expression upon pathogen or PAMP stimulation [13]. Few studies have examined the TLR mediated cytokine response at intestinal sites to ETEC infection [14–16]. In our previous study, IL-17A, IL-17B, IL-17F and other cytokines were detected in the intestinal tissues of F4^+^ETEC infected piglets, suggesting a role in host defense against infection [17]. However, despite its presence at the intestinal epithelium, it is unclear whether or how IL-17C is involved during acute ETEC infection. Moreover, there is no information on how IL-17C is induced by these pathogens.

Thus, in the present study, we addressed if an F4^+^ ETEC infection can induce IL-17C production in intestinal tissues/epithelial cells and determined whether and which type of TLR mediates the expression of IL-17C. In addition, we further demonstrated the role of IL-17C in epithelial host defense by analyzing the expression profile of host defense genes/proteins.

## Materials and methods

The methodology of the animal experiment was approved by the Ethical Committee of Jinhua Polytechnic (ECJHC2016-1010). Experiments were performed in accordance with the Regulations for the Administration of Affairs Concerning Experimental Animals approved by the State Council of the People’s Republic of China. F4 receptor-positive (F4R^+^) piglets (7–8 weeks old, Landrace) were selected and purchased from MEBOLO swine breeding Co., Ltd (Jinhua, China) based on the MUC4 TaqMan assay as previously described [18].

### Bacterial strains, cell line and culture conditions

The ETEC reference strain C83901 (O8:K87:F4ab, LT^+^, expression of flagellin, China Veterinary Culture Collection Center) and the non-pathogenic *E. coli* strain HB101 (no flagella, China General Microbiological Culture Collection Center) were cultured as previously described with minor modifications [19]. Briefly, the frozen bacteria were first recovered on a Brain Heart Infusion (BHI, Oxoid, Hampshire, UK) agar plate and then bacterial colonies of each strain were transferred into LB media and grown overnight with continuous agitation (180 rpm) at 37 °C. The porcine intestinal epithelial cell line IPEC-J2 (purchased from GuangZhou Jennio Biotech Co., Ltd, Guangzhou, China) was grown in Dulbecco’s Modified Eagle’s Medium/F12 (DMEM/F12 1:1; Thermo Fisher Scientific, Waltham, MA, USA) supplemented with 5% fetal calf serum (FCS, Sijiqing Biotech, Hangzhou, China), 2 mM l-glutamine (Thermo Fisher Scientific), 1 × Insulin/Transferrin/Selenium (ITS, Sigma-Aldrich, St. Louis, MO, USA), 100 U/mL penicillin and 100 μg/mL streptomycin (Thermo Fisher Scientific) and 5 ng/mL human epidermal growth factor (EGF, Thermo Fisher Scientific) at 37 °C and 5% CO_2_ in T75 cell culture flasks in a humidified atmosphere. These cells were seeded in a 24-well cell culture plate (5 × 10^5^ cells/well) and maintained in IPEC-J2 culture medium for up to 72 h until 100% confluent. Next, the culture medium was replaced with IPEC-J2 culture medium without FSC (differentiation medium) and further cultured for 48 h to induce differentiation.

### Small intestinal segment perfusion (SISP)

Three piglets were used to analyze the early immune response after ETEC infection. SISP experiments were performed essentially as previously described [19]. Three small intestinal segments from mid-jejunum were randomly perfused with 2.5 × 10^9^ CFU of the bacterial strain C83901, HB101 or PBS. In total, each segment was perfused with 32 mL of perfusion fluid over 4 h by injecting 2 mL every 15 min. Then, piglets were euthanized with an overdose sodium pentobarbital (Aspen, Wuhan, China) and a small piece of tissue of each segment was sampled, embedded in RNA later (Sigma-Aldrich), and stored at room temperature until RNA isolation.

### Bacterial inoculum and treatments

Prior to bacterial inoculation, IPEC-J2 monolayers were gently washed three times with sterile PBS and cultured in differentiation medium without antibiotics for 2 h at 37 °C, 5% CO_2_, 90% humidity. Then, the cells were inoculated with different bacterial strains (5 × 10^7^ CFU/well, MOI = 100) in antibiotic-free differentiation medium and incubated at 37 °C, 5% CO_2_ in a humidified atmosphere. After 1 h of incubation, cells were lysed in 1 mL TRIzol Reagent (Thermo Fisher Scientific) immediately after aspiration of the culture medium or further cultured for 1 h, 3 h and 24 h in differentiation medium supplemented with 50 μg/mL gentamycin (Thermo Fisher Scientific) after 3 times washing with PBS to remove non-adherent bacteria. Subsequently, cells were harvested and resuspended in TRIzol Reagent or directly lysed in RIPA lysis buffer (P0013, Beyotime, Shanghai, China) for Western blotting. The cell supernatants were collected and stored at −80 °C. To assess protein expression, cells on coverslips were fixed with 4% paraformaldehyde for 5 min at RT and stained with anti-IL-17C (1:1000, rabbit pAb, ab153896, Abcam, Cambridge, UK), anti-claudin-1 (1:500, rabbit pAb, 51-9000, Thermo Fisher Scientific) and anti-claudin-2 (1:300, mouse mAb, 32-5600, Thermo Fisher Scientific). Then, the sections were washed and incubated with the secondary antibody in different combination including FITC conjugated goat anti-rabbit IgG (H + L) (10 μg/mL, Thermo Fisher Scientific), Texas Red-X conjugated goat anti-mouse IgG (H + L) and FITC conjugated goat anti-mouse IgG (H + L) (10 μg/mL, Thermo Fisher Scientific) at RT for 1.5 h. The nuclei were counterstained with Hoechst (10 μg/mL, Sigma-Aldrich) and the slides were mounted in glycerol containing 0.223 M 1,4-diazobicyclo-(2,2,2)-octane (Sigma-Aldrich) and imaged on a confocal microscope (Leica TCS SP8 MP, Germany).

### Inhibitor, flagellin and cytokine treatments

Two hours before bacterial inoculation, IPEC-J2 monolayers were incubated with 0.5 μM oligodeoxyribonucleotide (ODN 2088, Miltenyi Biotec, Bergisch Gladbach, Germany; blocks TLR7/8/9 signaling as described previously [20, 21]), TH1020 (SML1741, Sigma-Aldrich, inhibits TLR5 signaling as described previously [22]), 20 or 40 ng/mL IL-17C (MyBioSource, San Diego, CA, USA) and then inoculated for 24 h with the different bacterial strains as described above. As flagellin has been reported to induce IL-17C production in several epithelial cells [7, 23, 24], IPEC-J2 monolayers were also stimulated with flagellin (100 ng/mL, FLA-ST Ultrapure, Invivogen, San Diego, CA, USA) in the presence or absence of TH1020. Then, the cells and/or culture supernatant were collected at 4 h and 24 h post stimulation, respectively. Subsequently, cells were harvested and resuspended in TRIzol Reagent or directly lysed in RIPA lysis buffer for Western blotting. The cell supernatants were collected and stored at −80 °C for later use.

As TH1020 and ODN 2088 were dissolved in DMSO and TE buffer, respectively, the toxic effects of these two solvents on IPEC-J2 monolayers were also investigated during the long incubation times using the propidium iodide (PI, Beyotime) assay. Briefly, IPEC-J2 monolayers were cultured at 24-well plates at a density of 5 × 10^5^ cells per well. The cells were then treated with TH1020 and ODN 2088 at 0.5 and 5 μM and incubated in 5% humidified carbon dioxide at 37 °C for 24 h. An aliquot of 1 μL of 500 μg/mL PI was added to each well and incubated for 60 min at room temperature. The fluorescence was measured using a multi-detection microplate reader (FLUOstar Omega, BMG LABTECH, Germany) with an excitation wavelength of 544 nm and an emission wavelength of 612 nm. In the first 60-min incubation period, measurements were taken at 15-min intervals to obtain a background level for the PI solution in the untreated cells. Then, measurements were taken at 30-min intervals for 23 h. At the end of the 24 h, EtOH (100%) was added to each well in order to induce maximal death, and the maximal PI fluorescence were taken afterwards. Cell viability determined using PI was calculated as a percentage of viable cells as follows:

$${\text{\% cell viability}} = 100{\text{\% }} - \frac{{\left[ {{\text{PIem of test }} - {\text{PIem of background}}} \right] \times 100{\text{\% }}}}{\text{PIem of maximum death level induced by EtOH}}$$

PIem = propidium iodide fluorescence emission, the experiment was carried out in 4 replicates on three independent batches of cells.

### Transepithelial electrical resistance assays

To further investigate the role of IL-17C on the barrier function of IPEC-J2 cells, collagen-coated 6-well transwell inserts were used to induce polarization of the monolayers (pore size 0.4 μm; surface area 4.67 cm^2^; Corning, New York, NY, USA) as described before [16]. Briefly, IPEC-J2 cells were seeded at a density of 5.0 × 10^5^ cells/well/mL on the transwell inserts and incubated in culture medium for 24 h. Then, the apical medium in the upper compartment was refreshed. The culture medium was refreshed every 2 days until the cells were roughly 80% confluent. Then, the medium was replaced by differentiation medium. To monitor differentiation, transepithelial electrical resistance (TEER) values were measured daily using the Millicell Electrical resistance system (Millipore, Billerica, MA, USA) until they reached 4000 Ω cm^2^.

Differentiated IPEC-J2 cells were subsequently incubated in triplicate either with the different bacterial strains (HB101 and C83901, 1.3 × 10^8^ CFU/transwell, MOI ≈ 100) or IL-17C (20 ng/mL) for 1 h at 37 °C in a humidified atmosphere. Then, the bacteria were removed by washing three times with sterile PBS. The IPEC-J2 monolayers were further incubated for another 1 h, 3 h and 23 h in differentiation medium supplemented with 50 μg/mL gentamycin. During this period, the epithelial integrity was determined by measuring the TEER every 2 h until 12 h post-incubation and at 24 h post-incubation. Cells for the different bacterial or IL-17C stimuli were taken after 2 h and 4 h post incubation and resuspended in TRIzol Reagent and stored at −80 °C for later use. Meanwhile, cells on the membranes were fixed and stained with anti-occludin (1:100, rabbit pAb, 71-1500, Thermo Fisher Scientific) for cellular visualization of this TJ in the differentiated IPEC-J2 cells as described above. The cell medium from the apical and basolateral compartment were collected at the end of the experiment and stored at −80 °C until further processing.

### RNA extraction and cDNA synthesis

RNA was isolated from intestinal tissues and IPEC-J2 cells using TRIzol Reagent as described [17]. The RNA concentration and purity was measured and calibrated using a NanoDrop Spectrophotometer (Wilmington, DE, USA) to an OD_260_/OD_280_ ratio of 1.8–2.0 and OD_260_/OD_230_ ratio of 1.9–2.1, and the RNA integrity was evaluated by agarose gel electrophoresis. Only high quality RNA was used for further processing. Total RNA (1 μg) was reverse transcribed using Superscript™ III Reverse Transcriptase (200 U; Invitrogen, Carlsbad, CA, USA) and random primers (7.5 ng/μL; Invitrogen). To check the synthesis of amplifiable cDNA in the reverse transcription, a conventional PCR step was performed using GAPDH, β-actin and RPL-19 specific primers (Table 1).

**Table 1:** **List of the primers used in the qPCR assay**

Gene	Sequence (5′ → 3′)	Size (bp)	Ta (°C)	References
IL-17A	F: ACTCCAAACGCTTCACCTCAC	234	58	[17]
	R: AGCCCACTGTCACCATCACTT			
IL-17C	F: CGTGTGGACACGGATGAGAG	217	60	Present study
	R: GGATGAACTCGGCGTGGAAG			
IL-17RE	F: CCCAGATTCCTCGCCATACC	106	60	Present study
	R: CCTGGCAACAGATACAGGCA			
TLR1	F: AGATTTCGTGCCACCCTATG	277	55	[50]
	R: CCTGGGGGATAAACAATGTG			
TLR2	F: TGCTATGACGCTTTCGTGTC	163	55	
	R: CGATGGAGTCGATGATGTTG			
TLR3	F: GAGCAGGAGTTTGCCTTGTC	149	55	
	R: GGAGGTCATCGGGTATTTGA			
TLR4	F: TCATCCAGGAAGGTTTCCAC	234	55	
	R: TGTCCTCCCACTCCAGGTAG			
TLR5	F: GGTCCCTGCCTCAGTATCAA	114	55	
	R: TGTTGAGAAACCAGCTGACG			
TLR6	F: TCAAGCATTTGGACCTCTCA	170	58	
	R: TTCCAAATCCAGAAGGATGC			
TLR7	F: TCTGCCCTGTGATGTCAGTC	317	55	
	R: GCTGGTTTCCATCCAGGTAA			
TLR8	F: CTGGGATGCTTGGTTCATCT	241	55	
	R: CATGAGGTTGTCGATGATGG			
TLR9	F: AGGGAGACCTCTATCTCCGC	205	55	
	R: AAGTCCAGGGTTTCCAGCTT			
TLR10	F: GCCCAAGGATAGGCGTAAAT	128	55	
	R: CTCGAGACCCTTCATTCAGC			
pBD-2	F: CTGTCTGCCTCCTCTCTTCC	168	60	[51]
	R: CAGGTCCCTTCAATCCTGTT			
Claudin-1	F: ACCCGCACTACGTCACCTTC	146	60	Present study
	R: GGCAGGACACCTGGTCATTG			
Claudin-2	F: AGAAGTTTCAAAGCCTGGGAG	119	60	[52]
	R: TCAACCTCATACAGGGGCAAA			
Occludin	F: GTGGTAACTTGGAGGCGTCTTC	102	58	[53]
	R: CCGTCGTGTAGTCTGTCTCGTA			
ZO-1	F: AAGGATGTTTACCGTCGCATT	253	60	
	R: ATTGGACACTGGCTAACTGCT			
TNF-α	F:GCATGGTGGTGGTTGTTTCTGACGAT	99	60	
	R:GCTTCTGTTGGACACCTGGAGACA			
IL-8	F: TCTCGGTGTAGAGCAAGG	146	58	
	R: TTCCCAAAGTGCTGGTATT			
β-Actin	F: TCATCACCATCGGCAACG	133	60	[54]
	R: TTCCTGATGTCCACGTCGC			
GAPDH	F: GGGCATGAACCATGAGAAGT	230	60	[55]
	R: AAGCAGGGATGATGTTCTGG			
RPL19	F: AACTCCCGTCAGCAGATCC	147	60	[56]
	R: AGTACCCTTCCGCTTACCG			

### Real-time qPCR

Primers (Table 1) were synthesized by Sangon Biotech (Shanghai, China). The amplification efficiency of all the reactions ranged from 94 to 103%. The PCR products were sequenced and subjected to agarose gel electrophoresis to verify their specificity. cDNA was diluted 8× in DEPC-treated ddH_2_O and combined with primer pairs and SYBR Green PCR Master Mix (Applied Biosystems, Warrington, UK) according to the manufacturer’s recommendations. Quantitative PCR (qPCR) assays were run on the StepOnePlus real-time PCR system (Applied Biosystems) with the following cycling conditions: 95 °C for 3 min, followed by 40 cycles of denaturation at 95 °C for 15 s, annealing for 30 s and elongation at 72 °C for 30 s. Fluorescence acquisition was measured at 72 °C and melting curve analysis was done at 65–95 °C with continuous fluorescence acquisition. GAPDH, β-actin and RPL-19 were selected as reference genes because of their stability [17]. All reactions were performed in triplicate and relative gene transcription values were calculated using the 2^−ΔΔCt^ method and normalized against these three selected reference genes [25].

### Western blot assays

The concentration of total protein in the cell lysates was determined with a bicinchoninic acid (BCA) protein assay kit (Thermo Fisher Scientific) according to the manufacturer’s instructions. Equal amounts of heat-denatured total protein (50 µg) in sample buffer were separated by SDS-PAGE (12%) and transferred onto PVDF membrane (Bio-Rad, Hercules, CA, USA) at 90 V for 45 to 60 min. After blocking with 5% non-fat milk powder in blocking buffer at room temperature for 1 h, membranes were incubated with primary antibody overnight at 4 °C. The primary antibodies used were: anti-β-actin (1:3000, rabbit pAb, ab8227, Abcam), anti-IL-17C (1:1000, rabbit pAb, ab153896, Abcam), anti-TLR5 (1:50, clone H127, Santa-Cruz Biotechnology, Santa Cruz, CA, USA), anti-porcine beta-defensin 2 (anti-pBD-2, rabbit pAb, CLOUD-CLONE CORP, Wuhan, China), anti-claudin-1 (1:250, rabbit pAb, 51-9000, Thermo Fisher Scientific), anti-claudin-2 (1:250, rabbit pAb, 51-6100, Thermo Fisher Scientific), anti-occludin (1:100, rabbit pAb, 71-1500, Thermo Fisher Scientific). After being washed extensively to eliminate aspecific binding, the membranes were incubated with a horseradish peroxidase-conjugated secondary antibody (anti-rabbit IgG, Santa-Cruz Biotechnology) at room temperature for 30 min. The antibody-reactive bands were visualized using chemiluminescence (Tanon 5200, Shanghai, China).

### Cytokine ELISA

The secretion of IL-17C in cell-free supernatants was measured using commercial ELISA kits (MyBioSource) according to the manufacturer’s guidelines. The IL-8 and TNF-α concentrations were assessed in both the apical and basolateral medium with porcine-specific ELISA kits (TNF-α: #PTA00; IL-8: # P8000; R&D Systems, Minneapolis, MN, USA) according to the manufacturer’s guidelines. All assays were performed in triplicate and the data are shown as mean ± SD.

### Statistical analysis

Statistical analysis was performed with the Mann–Whitney U test or Kruskal–Wallis test for the independent samples and Friedman’s Two-Way Analysis for the related samples in the SPSS 22 software package. The significance level was set at *p* < 0.05.

## Results

### F4^+^ ETEC induces IL-17C and TLR2, 5, 8 and -10 mRNA expression in small intestinal tissues

**Figure 1:**
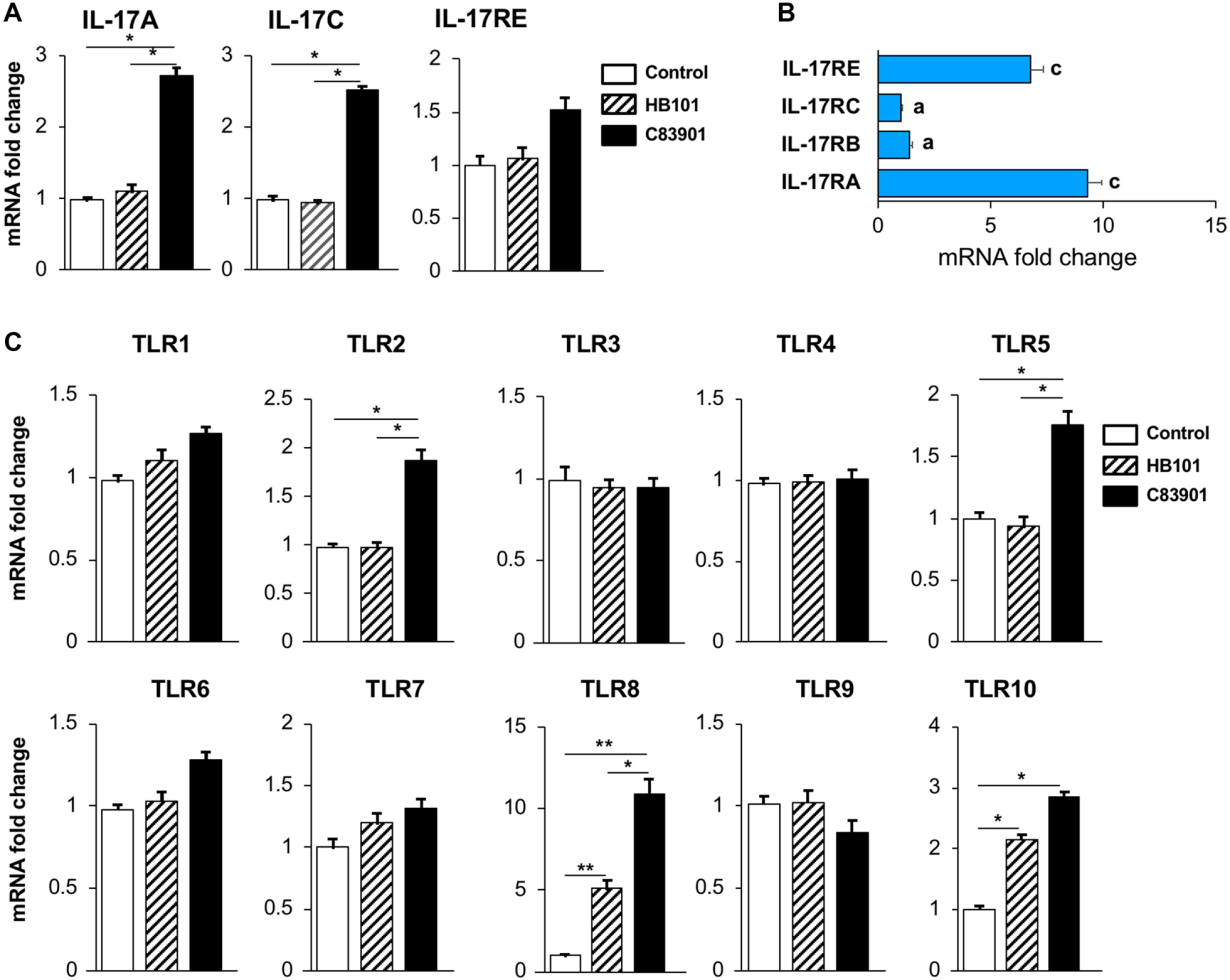
**F4**^**+**^ **ETEC induces IL-17C and TLR2, 5, 8 and -10 mRNA expression in small intestinal tissues.** Jejunal segments of three piglets were perfused with F4^+^ ETEC (C83901), non-pathogenic *E. coli* strain HB101 or PBS for 4 h. **A** The mRNA expression of IL-17A, IL-17C and IL-17RE in the intestinal segments was analyzed by qPCR. The mRNA expression level was normalized to the reference genes and then to the control group. Data are presented as the mean ± standard deviation (SD) (*n* = 3 per group). * *p* < 0.05. **B** The mRNA expression of different IL-17 receptors in jejunal segments in homeostatic conditions. The mRNA expression level was normalized to the reference genes and then to IL-17RC. Data are presented as the mean ± SD (*n* = 6), different letters indicate significant differences between groups (*p* < 0.01). **C** TLR mRNA expression profile in jejunal segments after F4^+^ETEC perfusion. Jejunal segments of three piglets were perfused with F4^+^ ETEC (C83901), non-pathogenic *E. coli* strain HB101 or PBS for 4 h. The mRNA expression of TLR-1 to 10 in the intestinal segments was analyzed by qPCR. The mRNA expression level was normalized to the reference genes and then to the control group. Data are presented as the mean ± SD (*n* = 3 per group). *p < 0.05, **p < 0.01.

Previously, we showed that the F4^+^ETEC strain GIS26 (O149:K91:F4ac^+^, LT^+^STa^+^STb^+^) elicited IL-17A mRNA expression in small intestinal tissues [19], so we first determined if another F4^+^ETEC strain, C83901 (O8:K87:F4ab, LT^+^, STb^+^), can also induce a similar response. Here, we found that C83901 induced IL-17A mRNA expression in the jejunal segments (Figure 1A). Similarly, IL-17C transcripts were also increased post challenge (Figure 1A), while no difference was found in the tissues perfused with the nonpathogenic *E. coli* strain HB101. The mRNA expression of the IL-17C receptor IL-17RE was not significantly altered by either HB101 or C83901 perfusion. However, jejunal segments displayed high constitutive IL-17RA and IL-17RE mRNA levels as compared to the other receptor subunits of IL-17 cytokines (IL-17RD not detected) (Figure 1B).

Given that IL-17C expression has been associated with TLR signaling [7, 23, 26], we determined the mRNA expression of all porcine TLRs from TLR1 to TLR10 upon ETEC perfusion. C83901 significantly increased TLR2, TLR5, TLR8 and TLR10 mRNA levels in the jejunal tissues as compared to control samples (Figure 1C). In contrast, HB101 perfusion only upregulated TLR8 and TLR10 transcript levels as compared to control samples (Figure 1C).

### F4^+^ ETEC induces IL-17C production and TLR5 and -8 mRNA expression by IPEC-J2

**Figure 2:**
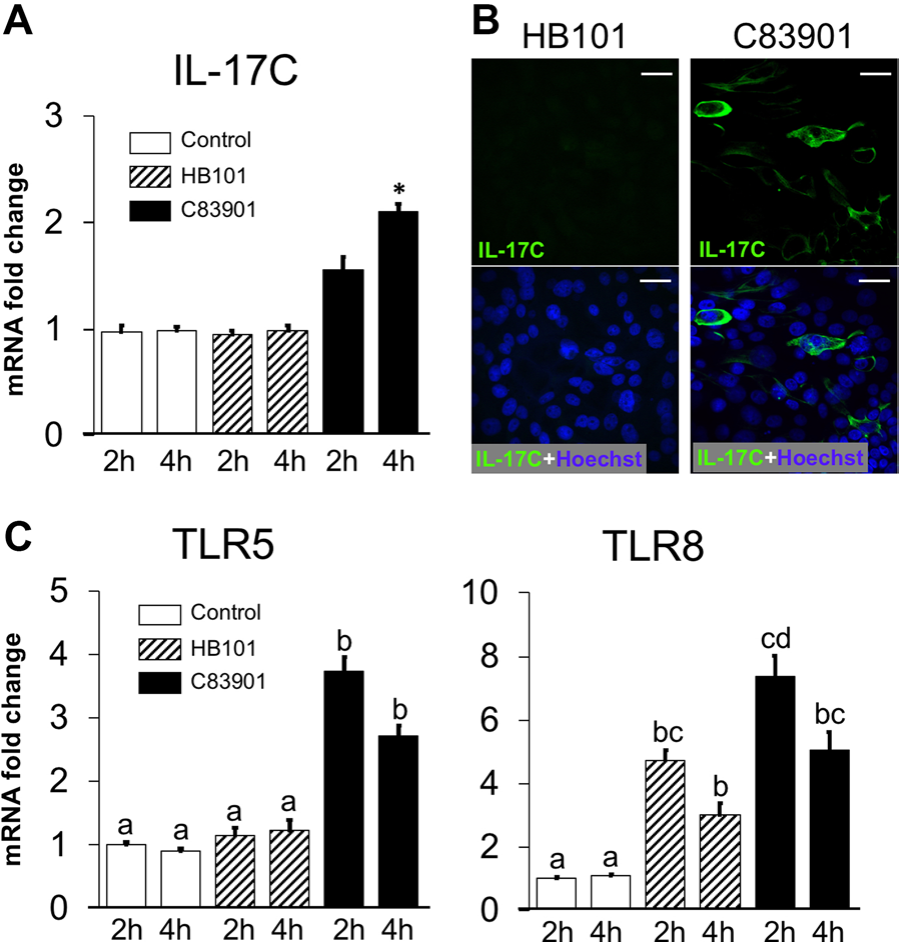
**Induction of IL-17C and TLR-5, -8 in the IPEC-J2 cells after F4**^**+**^**ETEC infection.** IPEC-J2 monolayers were inoculated with F4^+^ ETEC (C83901), non-pathogenic *E. coli* strain HB101 at MOI 100 or PBS for 1 h and further incubated for another 1 h, 3 h or 23 h, respectively. The mRNA expression of IL-17C (**A**) and TLR5 and TLR8 (**C**) in the IPEC-J2 monolayers was assessed by qPCR. The mRNA expression level was normalized to the reference genes and then to the control group of 2 h treatment. Data are presented as the mean ± SD (*n* = 3 per group), different letters indicate significant differences between groups (*p* < 0.05). **B** IPEC-J2 monolayers were inoculated with the different bacterial strains at MOI 100 for 1 h and then further incubated for 23 h. The cells were stained with anti-IL-17C (FITC, green). The nuclei were counterstained with Hoechst and representative confocal images are shown (*n* = 3). Scale bar = 25 μm.

As it was previously reported that IL-17C is preferentially expressed in IECs [7], we assessed if this IL-17C induction in the jejunum after C83901 infection also comes from the IECs. Thus, we used the cell line IPEC-J2 (originally isolated from the jejunum of a neonatal piglet) to study the IL-17C response after ETEC infection. Similar to the small intestinal tissues, only C83901 but not HB101 induced IL-17C mRNA expression in IPEC-J2 cells 4 h after infection (Figure 2A). This upregulated IL-17C expression was also confirmed at the protein level (Figure 2B). In addition, we also assayed the mRNA expression of TLRs in IPEC-J2 cells upon ETEC infection. Compared to the SISP model, significant changes were only observed for TLR5 and TLR8 mRNA expression by IPEC-J2 cells after C83901 infection, while upregulated TLR8 transcripts were also found in HB101 stimulated IPEC-J2 cells (Figure 2C). In line with the SISP model, the mRNA expression of TLR1, -3, -4, -6, -7 (not detected), and -9 was unaffected by either HB101 or C83901 infection in IPEC-J2 cells (Additional file 1). TLR2 and -10, which increased in the intestinal tissues, remained unchanged in IPEC-J2 cells following either HB101 or C83901 stimulation (Additional file 1).

### Blocking TLR5 inhibits F4^+^ ETEC induced IL-17C expression by IPEC-J2 monolayers

**Figure 3:**
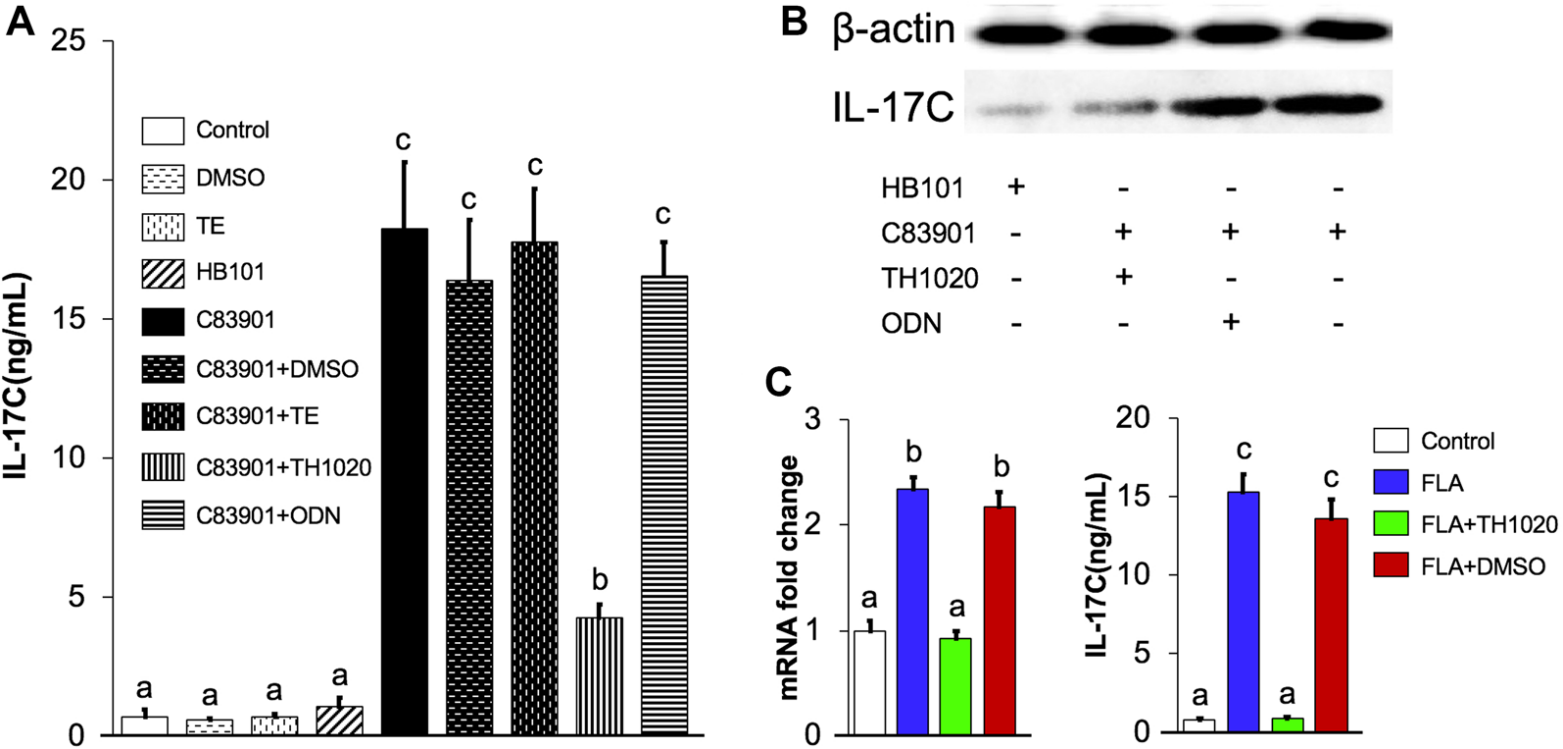
**F4**^**+**^**ETEC infection elicits an IL-17C response under the control of TLR5 in IPEC-J2 cells.** IPEC-J2 monolayers were first pre-cultured with 0.5 μM TH1020 (0.5 μM), 0.5 μM oligodeoxyribonucleotide (ODN) 2088 or the equivalent amount of their solvent DMSO and TE or PBS for 2 h. Then, the monolayers were inoculated with the C83901 at MOI 100 for 1 h, washed and further incubated for 23 h. **A** IL-17C cytokine production was analysed in the cell culture supernatant by ELISA. Data are presented as the mean ± SD (*n* = 5 per group). Different letters indicate significant differences between groups (a:b, *p* < 0.05; a:c, *p* < 0.01). **B** IL-17C protein was detected by Western blotting. **C** IPEC-J2 monolayers were first pre-cultured with TH1020 (0.5 μM, green bars), DMSO (red bars) or PBS (blue bars) for 2 h. Then, the monolayers were stimulated with flagellin (FLA, 100 ng/mL) and further incubated for 4 h and 24 h for mRNA and protein expression of IL-17C, respectively. Control samples only treated with PBS were marked with open bars. Different letters indicate significant differences between groups (a:b, *p* < 0.05; a:c, *p* < 0.01). Data are presented as the mean ± SD (*n* = 5 per group).

As the upregulation of TLR 5 and -8 correlated with the increase in IL-17C production in both the porcine intestinal tissues and IPEC-J2 cells upon F4^+^ ETEC stimulation, in a next effort we wanted to determine which TLR controls IL-17C expression in IECs upon ETEC infection. Given the fact that TLR5 engagement elicits the expression of IL-17C in human IECs [23], we further elucidated IL-17C expression in the presence of the TLR5 signaling inhibitor TH1020 [22] and oligodeoxyribonucleotides (ODN) 2088, which inhibit TLR7/8/9 signaling [20, 21]. As TH1020 and ODN 2088 were dissolved in DMSO and TE buffer, respectively, the effect of these two solvents on IL-17C production by IPEC-J2 monolayers was also investigated. DMSO and TE buffer had no effect on the induction of IL-17C mRNA (Additional file 2A) or protein in IPEC-J2 cells in the absence and presence of C83901 (Figure 3A). Moreover, the cell viability was unaltered by either TH1020 or ODN 2088 stimulation (Additional file 2B). However, we observed that IL-17C secretion was almost completely abolished by TH1020 in C83901 infected IPEC-J2 cells, showing the important role of TLR5 signaling in triggering IL-17C production by IPEC-J2 cells upon F4^+^ ETEC infection (Figures 3A and B). In contrast, no significant change in IL-17C expression was found with ODN 2088 incubation (Figures 3A and B), indicating that the activation of TLR8 signaling after ETEC infection is not responsible for the upregulated IL-17C production in IPEC-J2 cells.

As mentioned above, upon flagellin recognition TLR5 signaling triggers IL-17C production in IECs [7, 23, 24]. Considering flagellin is only produced by C83901 but not by HB101, we made an attempt to ascertain the involvement of flagellin in the TLR5–IL-17C axis. As shown in Figure 3C, a significant upregulation of IL-17C was observed in flagellin stimulated IPEC-J2 cells, which was completely inhibited by the presence of TH1020. These results indicate the direct role of flagellin in regulating the TLR5 mediated IL-17C induction in IPEC-J2 cells during C83901 infection.

### IL-17C regulates the intestinal epithelial response to ETEC infection

**Figure 4:**
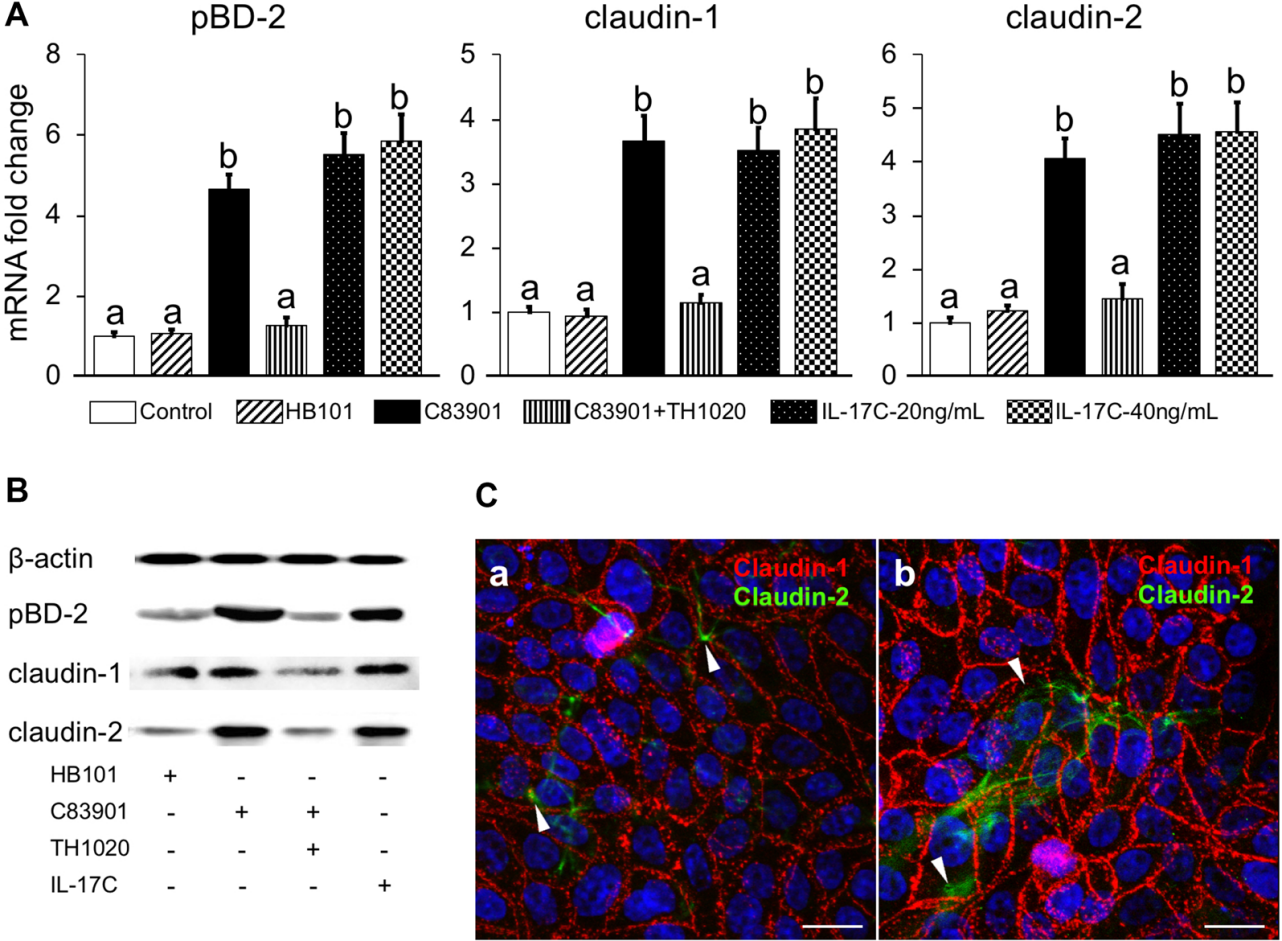
**TLR-5 mediated IL-17C induce pBD-2, claudin-1 and -2 expression in IPEC-J2 cells.** IPEC-J2 monolayers were pre-incubated with 0.5 μM TH1020 or the equivalent amount of its solvent DMSO for 2 h. Then, the monolayers were inoculated with HB101 or C83901 at MOI 100 for 1 h, washed and further incubated for 23 h. Non-treated cells were supplemented with PBS or IL-17C (20 or 40 ng/mL). **A** pBD-2, claudin-1 and -2 mRNA expression in the IPEC-J2 monolayers was assessed by qPCR. The mRNA expression level was normalized to the reference genes and then to the control group. Different letters indicate significant differences (*p* < 0.05). Data are presented as the mean ± SD (*n* = 5 per group). **B** pBD-2, claudin-1 and -2 protein were detected by Western blotting. IPEC-J2 monolayers were pre-incubated with TH1020 (0.5 μM) or the equivalent amount of its solvent DMSO for 2 h. Then, the monolayers were inoculated with HB101 and C83901 at MOI 100 for 1 h, washed and further incubated for 23 h. Non-treated cells were supplemented 20 ng/mL IL-17C. **C** Cellular localization of claudin-1 and -2 in IPEC-J2 cells 24 h post PBS (left panel) and C83901 (right pane, MOI 100) stimulation. The cells were stained with anti-claudin-1 (Texas-Red, Red), anti-claudin-2 (FITC, green). The nuclei were counterstained with Hoechst and representative confocal images are shown (*n* = 3). Scale bar = 10 μm.

It was previously reported that IL-17C could induce some epithelial cells to express genes that are involved in innate immunity, such as cytokines, chemokines, inflammatory mediators, and antimicrobial peptides [7–9]. To confirm the function of IL-17C on porcine intestinal epithelial cells, IPEC-J2 monolayers were treated with different bacterial strains and harvested for gene expression analysis. As TH1020 was demonstrated to be effective in blocking the TLR5-mediated IL-17C production in IPEC-J2 cells after ETEC infection, TH1020 and IL-17C were also added to the cells as controls. As shown in Figure 4A, C83091 infection stimulated pBD-2, claudin-1 and claudin-2 mRNA expression in IPEC-J2 cells. Adding exogenous IL-17C to the cells replicated these findings. As expected, blocking TLR5 with TH1020 dramatically inhibited pBD-2, claudin-1 and -2 mRNA expression in C83091 infected IPEC-J2 cells. The protein expression of these three genes was also examined by Western blotting and further confirmed the role of IL-17C and TLR5 signaling in the induction of host defense by IPEC-J2 cells upon ETEC infection (Figure 4B). The differential expression levels of claudin-1 and claudin-2 in ETEC infected IPEC-J2 cells were further analyzed by confocal microscopy. Both claudin-1 and -2 were increased in ETEC infected IPEC-J2 cells: claudin-1 is ubiquitously expressed and localized at tight junctions, while claudin-2 seemed only to be expressed by a subset of cells and was not evenly distributed over the plasma membrane (Figure 4C). Moreover, claudin-2 mostly localized adjacent to tight junctions in uninfected cells (upturned arrowheads, Figure 4C, left panel), while in ETEC infected IPEC-J2 cells its localisation shifted to the cytoplasma (downwards arrowheads, Figure 4C, right panel).

### IL-17C directly regulates inflammatory cytokine response and epithelial permeability in IPEC-J2 cells

**Figure 5:**
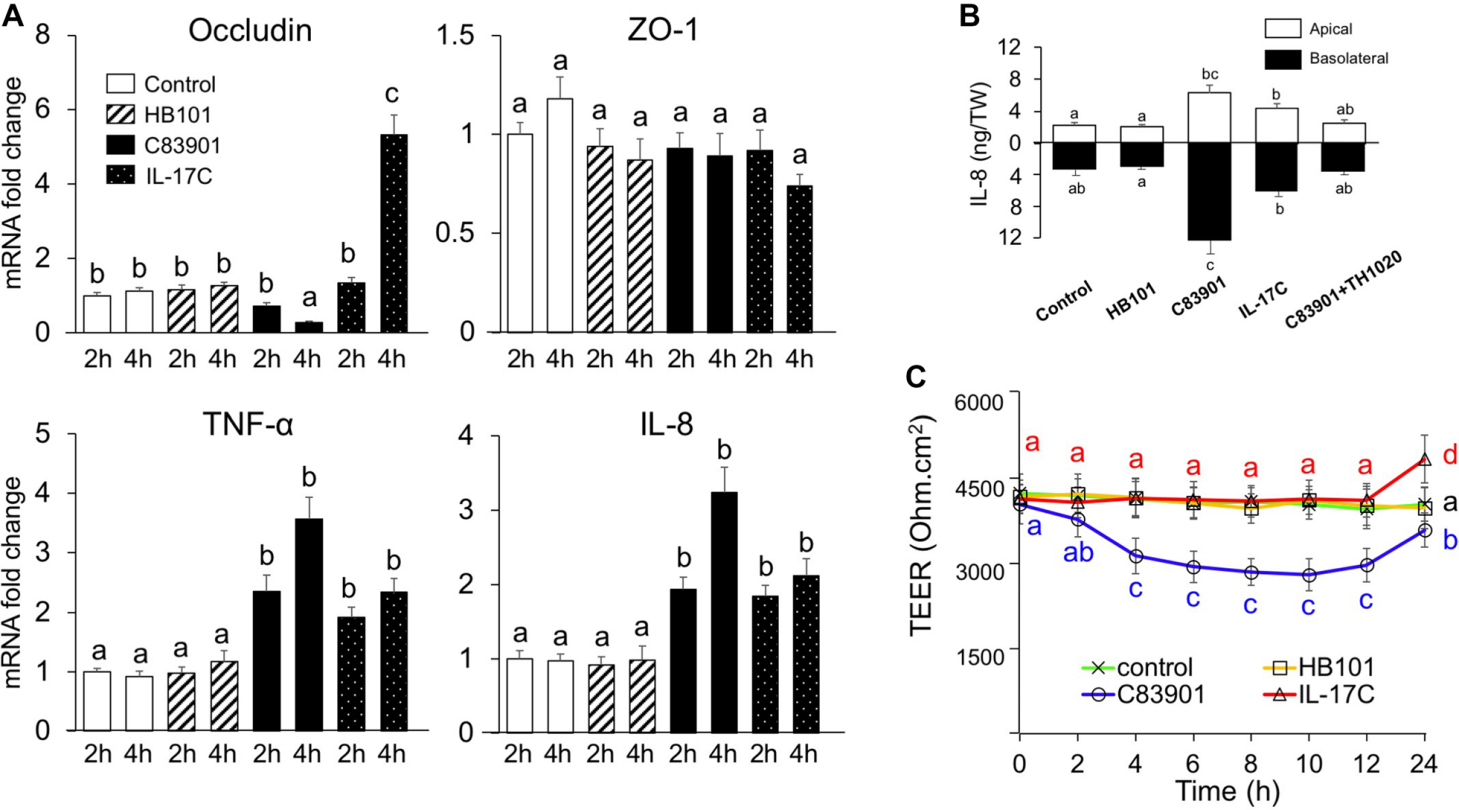
**Gene expression of tight junctions and proinflammatory cytokines and epithelial permeability changes after bacterial infection.** IPEC-J2 cells were cultured and differentiated on transwell inserts and then incubated with HB101 and C83901 at MOI 100 or PBS and IL-17C (20 ng/mL) for 1 h, washed and further incubated for another 23 h. **A** Occludin, ZO-1, TNF-α and IL-8 mRNA expression was assessed by qPCR in the IPEC-J2 monolayers at 2 and 4 h post-infection/stimulation. The mRNA expression level was normalized to the reference genes and then to the control group. **B** Apical and basolateral IL-8 secretion by differentiated IPEC-J2 cells 24 h post-infection/stimulation. IPEC-J2 monolayers were pre-incubated with TH1020 (0.5 μM) for 2 h. Then, the monolayers were inoculated with HB101 and C83901 at MOI 100 or PBS and IL-17C (20 ng/mL) for 1 h, washed and further incubated for 23 h. **C** Transepithelial electrical resistance (TEER) was measured in IPEC-J2 cells after bacterial infection or IL-17C stimulation. Different letters indicate significant differences (*p* < 0.05). Data are presented as the mean ± SD (*n* = 5 per group).

**Figure 6:**
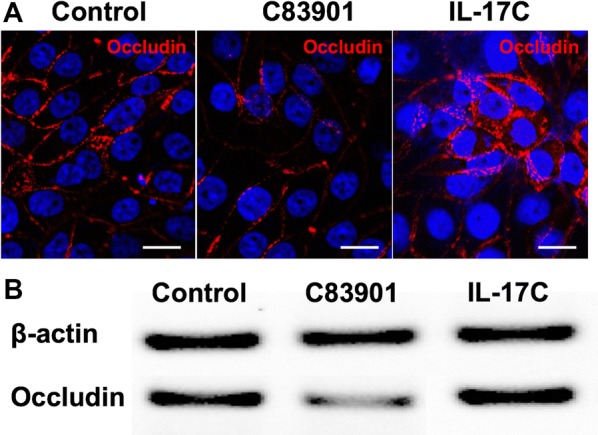
**IL-17C induce occludin expression in IPEC-J2 cells.** IPEC-J2 cells were cultured and differentiated on transwell inserts and then incubated with C83901 at MOI 100 or PBS and IL-17C (20 ng/mL) for 1 h, washed and further incubated for another 23 h. **A** Cellular localization of occludin in IPEC-J2 cells 24 h post C83901 and IL-17C stimulation. The cells were stained with anti-occludin (Texas-Red, Red) and the nuclei were counterstained with Hoechst and representative confocal images are shown (*n* = 3). Scale bar = 10 μm. **B** Occludin protein was detected by Western blotting in IPEC-J2 cells 24 h post C83901 and IL-17C stimulation.

Since it has been shown that inflammation can influence the expression of tight junction proteins (TJs), such as claudin-1, occludin and zonula occludens (ZO-1) [27, 28], we also assayed the mRNA expression of IL-8, TNF-α, occludin and ZO-1 by polarized IPEC-J2 cells using transwell inserts. As shown in Figure 5A, both C38901 infection and IL-17C stimulation upregulated IL-8 and TNF-α transcripts in IPEC-J2 cells as compared to control or HB101 treated cells. The induced secretion of IL-8 was also confirmed by ELISA in both the apical and basolateral medium (Figure 5B). However, TNF-α was not detected in culture media collected from both apical and basolateral sides of the cells under all of these experimental conditions (data not shown). In addition, C83901 infection decreased occludin mRNA expression in IPEC-J2 cells at 4 h post stimulation, while IL-17C exerted the opposite effect (Figure 5A). The effects of C83901 infection and IL-17C on occludin protein expression were further confirmed by an immunofluorescence assay (Figure 6A) and Western blotting (Figure 6B). In contrast to occludin, ZO-1 mRNA expression was not affected by IL-17C treatment or F4^+^ ETEC infection (Figure 5A).

To further confirm the role of IL-17C on the barrier integrity of IPEC-J2 cells, the TEER was measured during the first 24 h upon bacterial infection or IL-17C stimulation. The TEER in IPEC-J2 cells remained stable in the first 12 h, but increased significantly at 24 h in the presence of IL-17C. Infection with C83901 on the other hand significantly decreased TEER values as compared to control or HB101 treated monolayers from 4 h to 24 h (Figure 5C). Notably, TEER values significantly increased at 24 h as compared to 12 h after C83901 infection (Figure 5C).

## Discussion

Accumulating evidence has suggested that the epithelial expression of IL-17C can be induced directly by bacteria or indirectly through inflammatory cytokines [7, 8, 26]. The rapid kinetics of IL-17C expression following these stimuli positions IL-17C as an early player in the epithelial response to bacterial challenge [1]. TLR2, TLR4 or TLR5 signaling was shown to regulate IL-17C expression in human and mouse epithelial cells [7, 8, 23, 26, 29]. However, there have been no reports defining the IL-17C response in pigs. In the present study, we showed that F4^+^ ETEC infection triggered IL-17C production in small intestinal epithelial cells in a TLR5-dependent manner. This IL-17C controls the expression of antimicrobial peptides and tight junction proteins by these epithelial cells. This corroborates previous findings showing that TLR5 signaling triggers IL-17C production in human colonic epithelial cells [7, 23]. Flagellin is the only known ligand of TLR5 to date and has been shown to induce potent IL-17C production in several epithelial cells [7, 23, 24]. Here, we further demonstrated the involvement of flagellin in the TLR5 mediated IL-17C induction in porcine IECs. Although flagellin-dependent TLR5 signaling can be selectively inhibited by TH1020 [22], complete inhibition of IL-17C expression by TH1020 was only observed in flagellin, but not in F4^+^ ETEC stimulated cells. These results indicate that, in addition to TLR5 signaling, other pathways also might contribute to the IL-17C production during F4^+^ETEC infection.

F4^+^ETEC infection not only upregulated TLR5 mRNA expression in IECs, but also TLR8 expression. TLR8 recognizes single stranded RNA and was recently shown to control the potent immunogenicity of live attenuated vaccines by sensing microbial viability [30]. This might explain why the HB101 strain also triggered TLR8 mRNA expression, albeit to a lesser extent than F4^+^ ETEC. The upregulation of TLR2 in intestinal tissues and in IECs (slight increase) are in line with previous results, and probably serve as a signal to augment intestinal epithelial barrier integrity after F4^+^ETEC infection [31, 32]. Both F4^+^ETEC and ETEC-derived flagellin are known to trigger the fast secretion of pro-inflammatory cytokines by porcine IECs at the early stage of infection, such as TNF-α, prostaglandin E2 (PGE2), IL-6, IL-8 and IL-1α [16, 31, 33, 34], which are intended to disarm or destroy invading bacteria. Both *E. coli* strains induced TLR10 mRNA expression in intestinal tissues. TLR10 is expressed on immune cells [35], and although no ligands have yet been identified, this TLR seems to have an anti-inflammatory role [36]. Thus, increased TLR10 in the intestinal tissues is probably required to limit overwhelming inflammation to avoid inflammatory injury. However, an opposite result was found regarding the expression of these TLRs in the jejunum of diarrheal piglets [37], indicating that the TLRs expression profile also relates to the course of infection and disease severity after ETEC infection.

ETEC infection may increase the epithelial permeability by reducing the expression of tight junction proteins (TJs), such as claudin-1, in porcine IECs [38, 39], thus allowing the passage of electrolytes and water through the paracellular space and triggering diarrhea. However, here we found that ETEC strain C83901 increased both the mRNA and protein expression of claudin-1 and -2 in IPEC-J2 cells (at least at the 24 h time point after ETEC infection). In addition, the expression of these two genes can be also induced by exogenous IL-17C. Moreover, interference of IL-17C production through the TLR5 signaling inhibitor TH1020 decreased claudin-1 and -2 expression. These results indicate the participation of TLR5 mediated IL-17C in the induction of these claudins. Recently, a comprehensive study also detected the protein expression of several claudins (claudin-1, -3, -4, -5, -7, and claudin-8) in IPEC-J2 cells and no significant changes were observed between ETEC infected and control groups [33]. As those claudins were detected at 8 h post ETEC (IMT4818) infection, the inconsistent results in claudin expression might be attributed to the different sampling time or the different ETEC strains. Nevertheless, whether different F4^+^ETEC strains can differentially affect the expression of claudins needs further investigation. We also determined the subcellular localization of claudin-1 and claudin-2 and found that ETEC infection induces the cytoplasmic distribution of claudin-2, but not claudin-1. Claudin-1 are exclusively located in the junctional area of IPEC-J2 cells following infection with a variety of porcine ETEC strains and porcine epidemic diarrhea virus [40, 41], indicating the essential role of this TJ protein in preventing intestinal pathogens to enter the paracellular space and invade the host [42]. Claudin-2 has been shown to form a paracellular channel for small ions and water and its upregulation is linked to leaky epithelial barriers and diarrhea [43, 44]. Notably, this claudin-2-mediated epithelial permeability and diarrhea is critical to prevent intestinal pathology and to accelerate pathogen clearance [44]. The immunofluorescence microscopy results showed that ETEC infection increased cytoplasmic distribution and decreased the junctional localization of claudin-2. These findings may explain the upregulated expression of claudin-2 and declined TEER in IPEC-J2 cells after ETEC infection in the present study, and might indicate a role of claudin-2 in host defense rather than disease progression. In T84 human colon IECs, the enterotoxin STb can induce redistribution and/or fragmentation of ZO-1, claudin-1, and occludin due to actin rearrangement [45, 46]. Future studies addressing the effects of STb on tight junction structural proteins in porcine IECs would be worthwhile.

Additionally, we also determined the expression of the other two TJs including occludin and ZO-1 in IPEC-J2 cells. In agreement with other findings [31, 33, 39], ETEC infection significantly downregulated occludin expression at the mRNA level and at the protein level in the cell lysates. In contrast, we did not find any evident changes in the gene expression and protein expression or staining pattern (data not shown) of ZO-1 in F4^+^ETEC infected IPEC-J2 cells compared to those in control or HB101-treated cells. The impaired protein expression of ZO-1 has been reported in IPEC-J2 cells at 4 to 6 h post ETEC stimulation [31, 47]. Considering we investigated the protein expression profile only at 24 h post infection, we hypothesized that reduction of ZO-1 might occur at the early stage of infection. Downregulation of occludin following F4^+^ETEC infection is associated with a loss in TEER despite the upregulation of claudins. Interestingly, IL-17C significantly increased the expression of occludin and claudins in IPEC-J2 cells. Considering ETEC infection can induce the production of IL-17C, we speculate that IL-17C might participate in the regulation of the integrity of the intestinal barrier at a later stage of the bacterial infection. This is consistent with the significant increase in TEER at 24 h post ETEC infection or IL-17C stimulation (Figure 5C). Others have previously demonstrated the direct and protective role of IL-17C in regulating occludin production by colonic epithelial cells in mice during acute experimental colitis [48]. These results indicated that IL-17C is involved in mucosal barrier stability by regulating the production of claudin-1, -2, occludin and other TJs during ETEC infection.

In several human IECs, the increased expression of such claudins can be regulated by TNF-α and several inflammatory cytokines [27, 49]. Consistent with previous findings [16, 31, 33, 34], TNF-α and IL-8 were elicited in IPEC-J2 cells by F4^+^ETEC infection. Intriguingly, addition of exogenous IL-17C in IPEC-J2 cells also triggered the mRNA and protein expression of these two cytokines. Meanwhile, the TLR5 signaling inhibitor TH1020 exhibited a significant inhibition of C83901 induced IL-8 secretion. In the present study, TH1020 selectively inhibits the TLR5 mediated IL-17C production during ETEC infection. These results collectively suggests that the above mentioned claudins and other TJs are predominately regulated by IL-17C during ETEC infection. However, we cannot rule out an indirect role of TNF-α and other inflammatory cytokines in mediating TJs expression by epithelial cells as IL-17C has the capability of inducing fast inflammatory cytokines response in IECs [7].

Previous studies demonstrated IL-17C enhances the expression of mucosal host defense genes (hBD2, and S100 calcium-binding protein A12 (S100A12)) in an autocrine/paracrine manner in other epithelial cells [7, 24]. Interestingly, TLR5-mediated IL-17C production also induced the expression of pBD-2 in IPEC-J2 cells after F4^+^ ETEC infection. To our knowledge, we are the first to report that IL-17C induces the expression of antimicrobial peptides and tight junction proteins in porcine intestinal epithelial cells. In addition, exogenous IL-17C also increased the expression of pBD-2, claudin-1, claudin-2 and occludin expression. Given that the IL-17C receptor is constitutively expressed in IPEC-J2 cells, these results reveal a role of TLR5 mediated IL-17C in controlling barrier function and mucosal homeostasis in an autocrine/paracrine manner in the porcine intestine.

In summary, we have found that F4^+^ ETEC significantly induces the expression of IL-17C in porcine intestinal tissues, particularly in small intestinal epithelial cells, mainly mediated by flagellin-TLR5 engagement. We have further shown that IL-17C is sufficient to induce expression of antimicrobial peptides and tight junctions in IPEC-J2 cells, which indicates an essential role of IL-17C in mediating the innate immune response and barrier function in the intestine during F4^+^ ETEC infection.

## Additional files

**Additional file 1.** **mRNA expression profile of TLRs in the IPEC-J2 cells after HB101 or C83901 infection.** IPEC-J2 monolayers were inoculated with F4^+^ ETEC (C83901, black bars), non-pathogenic *E. coli* strain HB101 (diagonal stripe bars) at MOI 100 or PBS (open bars) for 1 h and further incubated for another 1 h and 3 h, respectively. The mRNA expression of TLR-1, -2, -3, -4, -6 -7, -9 and -10 in the IPEC-J2 monolayers was assessed by qPCR. The mRNA expression level was normalized to the reference genes and then to the control group of 2 h treatment. Data are presented as the mean ± SD (*n* = 3 per group), different letters indicate significant differences between groups (*p* < 0.05).

**Additional file 2.** **Effects of TH1020 and ODN 2088 or their solvents on IL-17C expression and cell viability in IPEC-J2 cells. (A)** DMSO and TE did not affect IL-17C mRNA expression in the IPEC-J2 cells. IPEC-J2 cells were stimulated with DMSO (1:1000) and TE (1:400) for 4 h. Cells incubated with PBS as control. The mRNA expression of IL-17C in the IPEC-J2 monolayers was assessed by qPCR. The mRNA expression level was normalized to the reference genes and then to the control group of 4 h treatment. Data are presented as the mean ± SD (*n* = 3 per group). **(B)** Cell viability was measured using the propidium iodide (PI) assay for the cytotoxic effects of TH1020 and ODN 2088. PI signals are expressed as a percentage death which is normalized against the resting level (0%) and the maximum death level induced by EtOH/Triton × (100%). The data are represented as the mean ± SEM of 4 replicates and are representative for three different experiments.
