# Supplementary figures and images for: Toll-like receptor 5-mediated IL-17C expression in intestinal epithelial cells enhances epithelial host defense against F4+ ETEC infection
Source: Vet Res. 2019 Jun 20;50:48. doi: 10.1186/s13567-019-0665-8 (PMC6584996; doi:10.1186/s13567-019-0665-8)

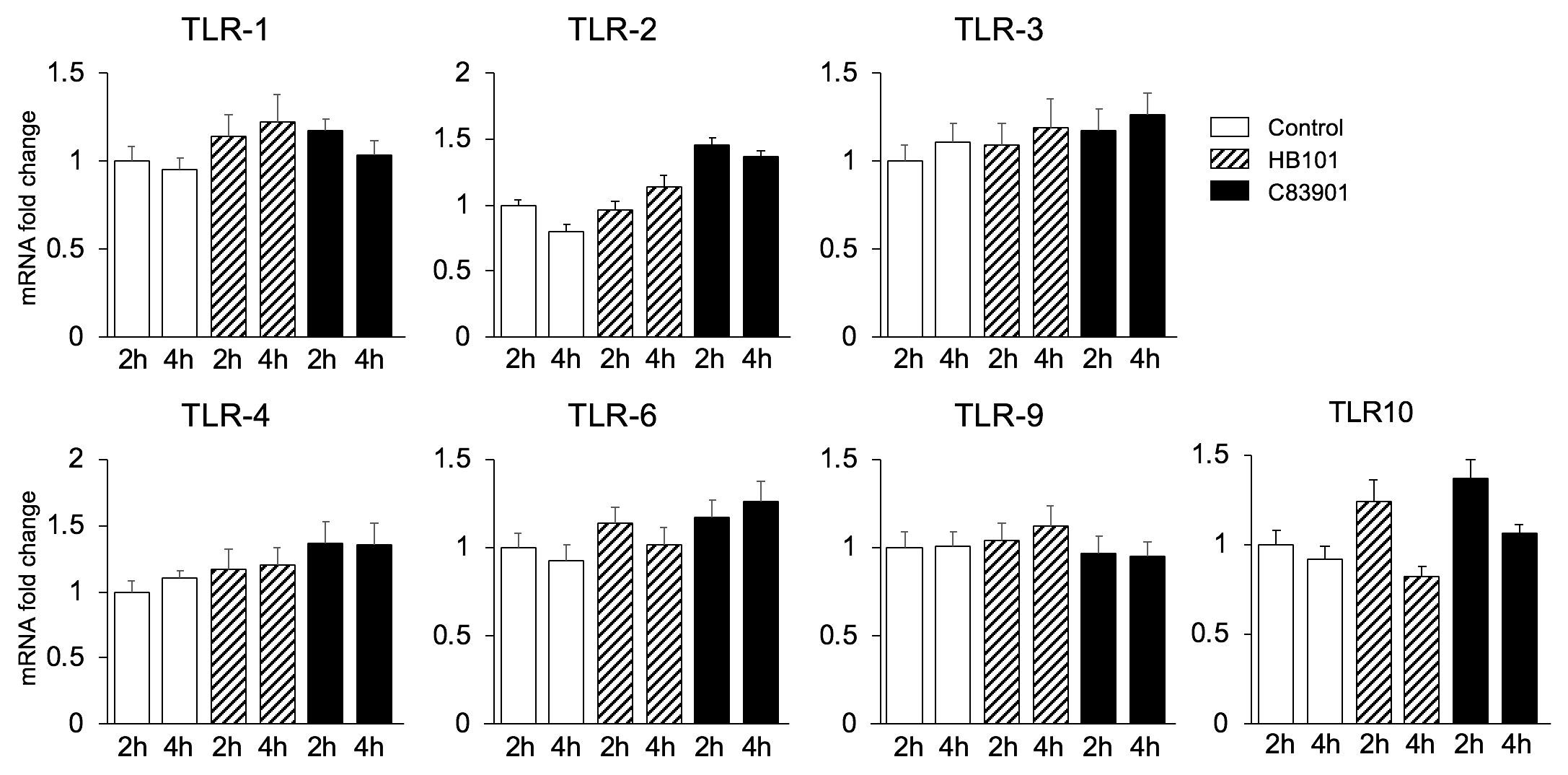

Supplement: Supplementary file 1 — Additional file 1. mRNA expression profile of TLRs in the IPEC-J2 cells after HB101 or C83901 infection. IPEC-J2 monolayers were inoculated with F4+ ETEC (C83901, black bars), non-pathogenic E. coli strain HB101 (diagonal stripe bars) at MOI 100 or PBS (open bars) for 1 h and further incubated for another 1 h and 3 h, respectively. The mRNA expression of TLR-1, -2, -3, -4, -6 -7, -9 and -10 in the IPEC-J2 monolayers was assessed by qPCR. The mRNA expression level was normalized to the reference genes and then to the control group of 2 h treatment. Data are presented as the mean ± SD (n = 3 per group), different letters indicate significant differences between groups (p < 0.05). [file 13567_2019_665_MOESM1_ESM.jpg]

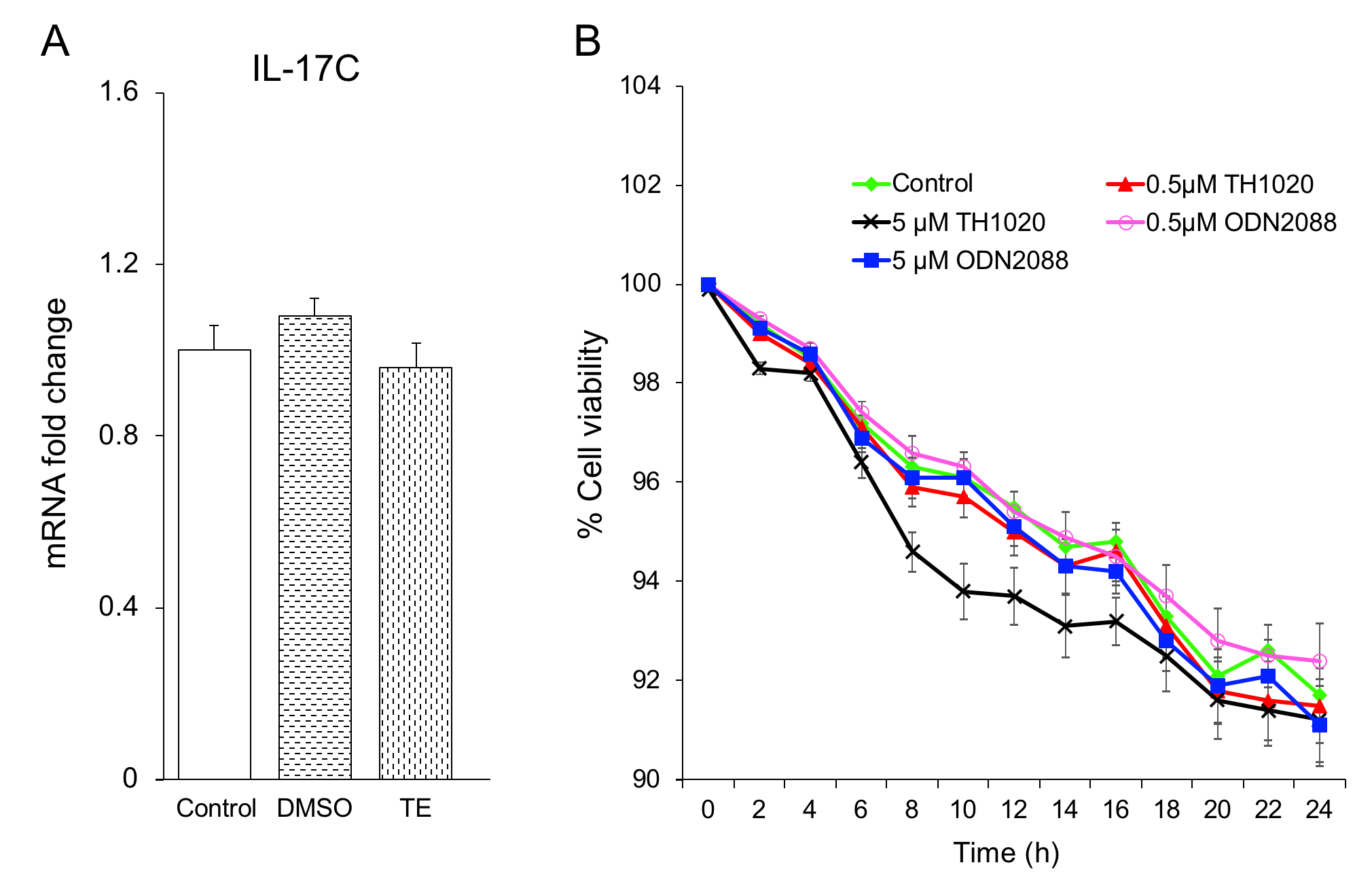

Supplement: Supplementary file 2 — Additional file 2. Effects of TH1020 and ODN 2088 or their solvents on IL-17C expression and cell viability in IPEC-J2 cells. (A) DMSO and TE did not affect IL-17C mRNA expression in the IPEC-J2 cells. IPEC-J2 cells were stimulated with DMSO (1:1000) and TE (1:400) for 4 h. Cells incubated with PBS as control. The mRNA expression of IL-17C in the IPEC-J2 monolayers was assessed by qPCR. The mRNA expression level was normalized to the reference genes and then to the control group of 4 h treatment. Data are presented as the mean ± SD (n = 3 per group). (B) Cell viability was measured using the propidium iodide (PI) assay for the cytotoxic effects of TH1020 and ODN 2088. PI signals are expressed as a percentage death which is normalized against the resting level (0%) and the maximum death level induced by EtOH/Triton × (100%). The data are represented as the mean ± SEM of 4 replicates and are representative for three different experiments. [file 13567_2019_665_MOESM2_ESM.jpg]
